# Retrospective Analysis of Antimicrobial Susceptibility of Uropathogens Isolated from Pediatric Patients in Tertiary Hospital at Al-Baha Region, Saudi Arabia

**DOI:** 10.3390/healthcare9111564

**Published:** 2021-11-17

**Authors:** Mohammed Abdullah Alzahrani, Heba Hassan Mohamed Sadoma, Sowmya Mathew, Saleh Alghamdi, Jonaid Ahmad Malik, Sirajudheen Anwar

**Affiliations:** 1 Pharmaceutical Care Department, King Fahad Hospital, Al Baha 65732, Saudi Arabia; malzahrani172@moh.gov.sa (M.A.A.); Hsadoma2010@hotmail.com (H.H.M.S.); 2 Department of Pharmacy Practice, East Point College of Pharmacy, Rajiv Gandhi University of Health Sciences, Bengaluru 560049, India; sowmyamathewk@gmail.com; 3 Department of Clinical Pharmacy, Faculty of Clinical Pharmacy, Al Baha University, Al Baha 65779, Saudi Arabia; saleh.alghamdi@bu.edu.sa; 4 Department of Pharmacology & Toxicology, National Institute of Pharmaceutical Education and Research (NIPER), Guwahati 781101, India; junaidpsst@gmail.com; 5 Department of Biomedical Engineering, Indian Institute of Technology (IIT), Ropar 140001, India; 6 Pharmacology & Toxicology Department, College of Pharmacy, University of Hail, Hail 81442, Saudi Arabia

**Keywords:** uropathogen, antibiotics resistance, pediatric patients, Al Baha, Saudi Arabia

## Abstract

Introduction: Prompt diagnosis and initiation of treatment are essential in preventing long-term renal scarring. However, increasing antibiotic resistance may delay the initiation of appropriate therapy. Methodology: A retrospective chart review was performed for patients admitted to the pediatric department with urinary tract infection (UTI) diagnosis in a large tertiary care hospital in Al Baha, Saudi Arabia, from May 2017 to April 2018. The study included children of both sexes under the age of 14 years. Results: Out of 118 urinary bacterial samples, *Escherichia coli* was the main etiologic agent in the community- and hospital-acquired infections. The infection rate was higher in girls (68.64%) than in boys (31.36%). The commonest isolates were *Escherichia coli* (44.07%), extended-spectrum beta-lactamase-producing *Escherichia coli* (11.86%), *Klebsiella pneumoniae* (9.32%), *Enterococcus faecalis* (7.63%), methicillin-resistant *Staphylococcus epidermidis* (4.24%), and coagulase-negative *Staphylococci* (3.39%). The current study demonstrates that nitrofurantoin (19%) was the most commonly prescribed medication in the inpatient and outpatient departments, followed by trimethoprim/sulfamethoxazole (16%), amoxicillin/clavulanic acid (15%), cefuroxime (10%), azithromycin (8%), ceftriaxone (7%), and ciprofloxacin (4%), while amikacin, amoxicillin, ampicillin, cefepime, imipenem, phenoxymethylpenicillin were prescribed less commonly due to the high resistance rate. Conclusion: The microbial culture and sensitivity of the isolates from urine samples should be routine before starting antimicrobial therapy. Current knowledge of the antibiotic susceptibility patterns of uropathogens in specific geographical locations is essential for choosing an appropriate empirical antimicrobial treatment rather than reliance on recommended guidelines.

## 1. Introduction

Urinary tract infection (UTI) is a bacterial infectious process affecting any part of the urinary tract, most commonly the bladder and the urethra. Urinary urgency and frequency, burning sensation during urination, lower abdominal discomfort, and turbid urine are the common symptoms of UTI. It is divided into lower (cystitis) and upper (pyelonephritis) UTI. Cystitis is less common in children below two years of age than pyelonephritis, whereas cystitis is much more common in adults than pyelonephritis [1]. UTI is common in children and is one of the leading causes of hospital admission in the pediatric population. About 3–8% of girls and 1–3% of boys experience a UTI before 14 years [1,2,3]. The diagnosis of UTI is complex in infants and young children, as urinary symptoms are very nonspecific. Fever may be the only symptom of UTI, especially in young children [2,4]. The delay in the diagnosis and treatment can lead to renal scarring, hypertension, and renal insufficiency, which may be the primary cause of increased morbidity rates in children.

On the contrary, overdiagnosis is responsible for unnecessary antibiotics, radiation exposure, and high cost [5,6]. Though the clinical history and physical examination are essential in the diagnosis of UTI, confirmation should be done by urine culture, which will demonstrate whether there is any proliferation of microorganisms in the urinary tract [4]. Therefore, before going for any antibiotic treatment, a urine culture must be performed. The most common uropathogens in pediatric UTIs are *Escherichia coli* (*E. coli*) bacteria, followed by *Proteus* spp., *Staphylococcus saprophyticus*, *Klebsiella* spp., and other *Enterobacteriaceae*. Antibiotics commonly recommended for UTIs treatment include co-trimoxazole (trimethoprim/sulfamethoxazole), nitrofurantoin, ciprofloxacin, and ampicillin. However, evidence suggests an existing relationship between extensive antimicrobial use and resistance, which ultimately limits the treatment options, hence resulting in more complications. For this reason, surveillance of antibiotic resistance is crucial for determining the pattern of antimicrobial resistance and consequently for guiding the selection of empirical therapy [6,7]. Therefore, this study aimed to determine the various bacteria causing UTI in pediatric patients and the antimicrobial resistance pattern isolated from pediatric patients at the tertiary hospital at Al Baha, Saudi Arabia.

## 2. Materials and Methods

### 2.1. Sample Size

This retrospective study was conducted from the test results of urine samples collected from patients admitted to King Fahad Hospital in the Al-Baha region, Saudi Arabia, from May 2017 to April 2018 (Protocol approval October 2020). A total of 118 patient files were assessed during the study period.

### 2.2. Inclusion Criteria

All pediatric patients between 0 and 14 years of age who were infected with UTI from May 2017 to April 2018 were included in the study.

### 2.3. Exclusion Criteria

Patients above 14 years of age were excluded from this study.

### 2.4. Data Collection

A unified and predesigned data collection form was designed to collect data from patients’ electronic medical records and laboratory reports with pre-specified data variables. The collected data were only used for the benefits of this study. We obtained the sex and age of patients, date of urine sample collections, bacterial isolate, antibiotics sensitivity/resistance, and antibiotics prescribed through the data collection form. The privacy and integrity of collected data were ensured.

#### 2.4.1. Urine Collection

For infants, babies, and young children, three methods of urine collection were followed. The first method was a sample that was acquired using the ‘clean catch method’, which involves placing the baby/child over a sterile receptacle. The second is the ‘bagged method’ in which a sterile perineal collecting bag is affixed to the perineal area, ensuring that the urethra is contained within the enclosed area. The third method is ‘suprapubic bladder aspiration’ and is only used as a last resort in the event of an emergency. For children above age 10, midstream urine was collected, or 20 mL urine was retrieved from the catheter with a sterile syringe into a sterile container after the initial urine was voided.

#### 2.4.2. Urine Analysis

Urine specimens were grown on blood agar/cystine lactose electrolyte deficient agar (CLED)/MacConkey agar plates and cultured for 24 to 48 h at 37 °C using a validated loop of 1 µL. The bacterial isolates were then Gram-stained and classified as Gram-positive cocci or Gram-negative rods. The VITEK system was used to perform comprehensive confirmation and antibiotic susceptibility tests. The collected data were examined and interpreted in accordance with the Clinical Laboratory Standards Institute’s criteria (CLSI).

### 2.5. Ethical Approval

The Scientific and Research Committee at King Fahad Hospital in Al Baha, Saudi Arabia, approved the study (Protocol dated 1 October 2020). The details and information gathered were kept confidential. This study did not contain any personal information. This was a secondary analysis of routine monitoring data that were anonymized.

### 2.6. Statistical Analysis

Data were entered into a database designed using an MS Excel spreadsheet and analyzed using Statistical Package for Social Sciences (SPSS Inc., Chicago, IL, USA) version 16. Study findings were explained in tables and graphs. Retrospective study statistics illustrate uropathogenic percentage and antibiotics resistance/sensitivity for particular periods. The overall 1-year resistance/sensitivity rates of the most common uropathogens to the routinely tested first-line antimicrobials were calculated by dividing the number of urinary isolates resistant/sensitive to each antimicrobial agent by the number of isolates tested against an individual antimicrobial agent. Tables and graphs are presented as frequency (count) and percentages with a 95% confidence interval.

## 3. Results

### 3.1. The Uropathogenic Percentage for the Entire Study Period

**Figure 1:**
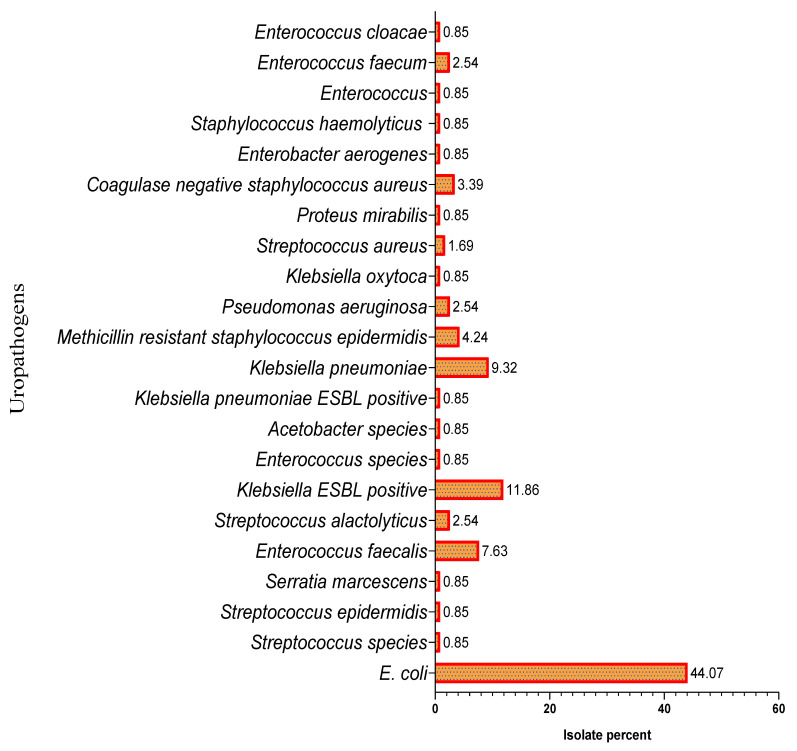
Percent of uropathogens from May 2017 to April 2018. Note: Total 118 uropathogen isolates were identified between May 2017 and April 2018; among them, four major UTI-causing bacteria were identified as *E. coli* = 52; ESBL-positive *E. coli* = 14; *K. pneumoniae* = 11; *E. faecalis* = 9.

**Figure 2:**
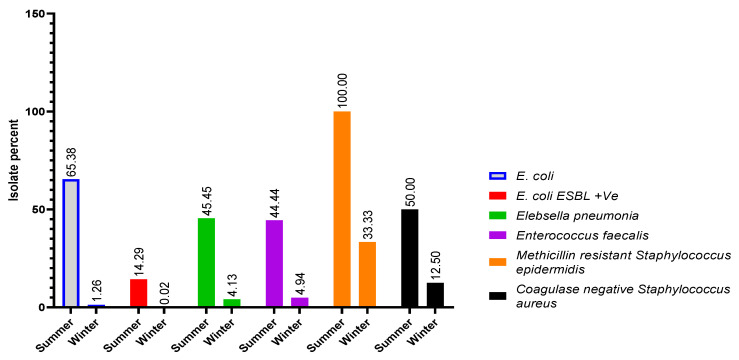
Percentage of uropathogens during summer and winter. **Note**: The 118 uropathogenic isolates were identified between May 2017 and April 2018; the summer season was categorized from March to August and September to February, which was considered winter season.

The pathogens causing UTI are well known, and *E. coli* was the leading etiologic agent in the community, as well as hospital-acquired infections. A total of 118 urinary bacterial samples were isolated and identified. The overall percentage of bacterial infections was higher in females (68.64%) than males (31.36%). The identified prevalent bacterial species were *E. coli*, extended-spectrum beta-lactamase-producing *Escherichia coli* (ESBL-positive *E. coli*), *Klebsiella pneumoniae* (*K. pneumoniae*), *Enterococcus faecalis* (*E. faecalis*), methicillin-resistant *Staphylococcus epidermidis* (MRSE), and coagulase-negative *Staphylococci* (CoNS), with the percentage population of 44.07%, 11.86%, 9.32%, 7.63%, 4.24%, and 3.39%, respectively. The demographic data of studied pediatric patients are shown in Table 1, and the percentage population of uropathogens isolated between May 2017 and April 2018 is demonstrated in Figure 1. The UTI was higher in summer, with 58.47%, than in winter, with 41.53%, as shown in Figure 2.

### 3.2. Antibacterial Susceptibility Pattern

The susceptibility pattern of *E. coli* was observed to be sensitive towards a broad range of antibiotics, such as cefotaxime, ceftazidime, colistin, levofloxacin, and tigecycline (100%), followed by nitrofurantoin (95.74%), amikacin (94.59%), imipenem (93.54%), aztreonam, and meropenem (88.88%), gentamicin (87.5%), tazocin (85.71%), ceftriaxone (75%), norfloxacin (75%), cefepime (71.4%), ciprofloxacin (68.18%), nalidixic acid (66.66%), and cefuroxime (53.33%). On the other hand, antibiotics such as amoxicillin/clavulanic acid (57.14%) and sulfamethoxazole/trimethoprim (55.55%) were less sensitive against *E. coli*. The resistance was observed with ampicillin (94.11%), cephalothin (92.30%), and cefoxitin (76.92%) in treated patients (Table 2).

The susceptibility patterns against ESBL-positive *E. coli* had a similar pattern as that shown by *E. coli*. They were highly sensitive to imipenem, meropenem, nitrofurantoin, and norfloxacin (100%), followed by amikacin and tazocin (92.31%) and ciprofloxacin (72.73%). Antibiotics such as amoxicillin/clavulanic acid, sulfamethoxazole/trimethoprim (66.67%), and gentamicin (63.64%) exhibited a lower susceptibility pattern against ESBL-positive *E. coli*. The antibiotics ampicillin, aztreonam, cefepime, cefoxitin, ceftazidime, ceftriaxone, cefuroxime, cephalothin, and levofloxacin demonstrated resistance. The first-line therapy for these types of bacteria consisted of nitrofurantoin, amoxicillin/clavulanic acid, and sulfamethoxazole/trimethoprim (Table 3).

*K. pneumoniae* was highly sensitive to amikacin, aztreonam, cefepime, ciprofloxacin, gentamicin, imipenem, and levofloxacin (100%), followed by meropenem, nitrofurantoin, and norfloxacin (80%), ceftriaxone (66.67%), tazocin (60%), and amoxicillin/clavulanic acid (50%). K. Pneumoniae was found highly resistant against ampicillin, cefoxitin, cefuroxime, cephalothin, and tobramycin (100%). At the same time, sulfamethoxazole/trimethoprim showed a lesser sensitivity pattern of 63.64% (Table 4).

*E. faecalis* was found highly sensitive to cephalothin, ciprofloxacin, linezolid, moxifloxacin, nitrofurantoin, teicoplanin, and vancomycin (100%) and sulfamethoxazole/trimethoprim (75%) and imipenem (66.67%), but it was highly resistant to cefepime and tetracycline (100%), followed by clindamycin and erythromycin (66.67%), cefoxitin and gentamicin (50%), and ampicillin (42.86%) (Table 5).

### 3.3. Antibiotics Prescribed for UTI

**Figure 3:**
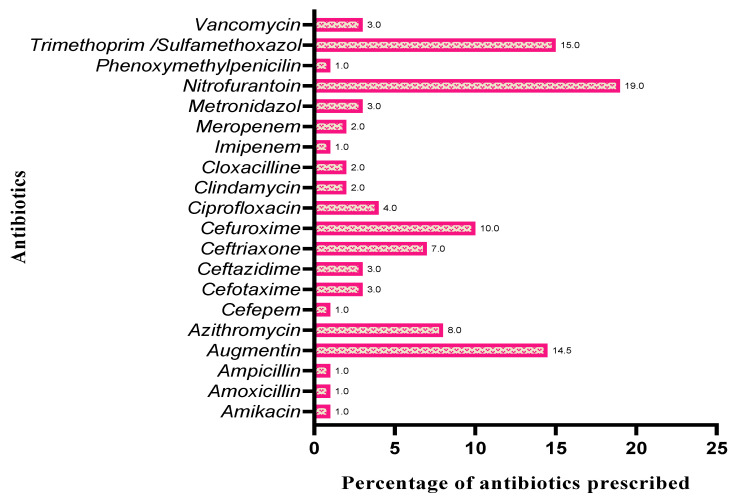
Percentage of antibiotics prescribed between May 2017 and April 2018.

The current study revealed that nitrofurantoin was the most prescribed medication in inpatient and outpatient departments (19%), and that it is highly sensitive against studied uropathogenic *E. coli*, *ESBL-positive E. coli*, *K. pneumoniae*, and *E. faecalis*; followed by trimethoprim/sulfamethoxazole (16%) against UTI caused by *E. coli*, *ESBL-positive E. coli*, and *E. faecalis*; amoxicillin/clavulanic acid (15%) against *E. coli*, *ESBL-positive E. coli*, and *K. pneumoniae*; cefuroxime (10%) against *E. coli*; azithromycin (8%) against *E.coli*; ceftriaxone (7%) against *E. coli* and *K. pneumoniae*; and ciprofloxacin (4%) against *E. coli*, *ESBL-positive E. coli*, *K. pneumoniae*, and *E. faecalis*. Amikacin, amoxicillin, ampicillin, cefepime, imipenem, and phenoxymethyl penicillin were found less prescribed because of resistance cases (Figure 3).

## 4. Discussion

The present study aimed to investigate the causative organisms frequently associated with UTI among the pediatric population in a tertiary care hospital in Al Baha in Saudi Arabia and to investigate the drug resistance profile of such infections. According to EAU guidelines on pediatric urology, UTI represents the most common bacterial infection in children caused by most frequent uropathogens such as *E. coli* (~75%), *K. pneumoniae*, *Enterobacter* spp., *Enterococcus* spp., *Pseudomonas* spp., and *Candida* spp. The guideline recommends that the choice of agent is also based on local antimicrobial sensitivity patterns and should later be adjusted according to sensitivity-testing of the isolated uropathogen. Frequently used antibacterial substances for UTI therapy in infants and children were cephalosporins, trimethoprim or trimethoprim/sulfamethoxazole, ampicillin, amoxicillin, amoxicillin/clavulanic acid, tobramycin, gentamicin, ciprofloxacin, and nitrofurantoin [8]. Our investigation also found a similar pattern of etiological uropathogens and antibacterial agents, with some uropathogen heterogeneity and antibacterial resistance. Ampicillin was found to be a highly resisted antibiotic against all major uropathogens.

In a meta-analysis of studies including children with UTI, a history of previous UTI and fever >40 °C were the two most helpful signs in identifying UTI in children below two years of age. Older children also present with fever, symptoms of the lower urinary tract, and abdominal pain, flank pain, chills, and fever are suggestive of pyelonephritis [9]. Abdominal pain, back pain, frequency and/or dysuria, and new-onset urinary incontinence were the most valuable signs in predicting UTIs in verbal children. In acute cystitis, children typically present with the absence of fever and symptoms from the lower urinary tract, including dysuria, frequency, urge, new-onset urinary incontinence, suprapubic/abdominal pain, and/or hematuria [10]. In our study, cystitis and pyelonephritis were primarily distinguished based on the history of symptoms. Acute cystitis is treated with nitrofurantoin, sulfamethoxazole/trimethoprim, amoxicillin/clavulanic acid, and oral cephalosporin [11]. According to our data, these drugs were the most usually prescribed, indicating that most of the cases investigated are related to pediatric cystitis.

In the present study, higher prevalence of UTI in girls (68.64%) than in boys (31.36%) was observed, which correlates with the findings of Hameed et al. (2019) showing that the frequency of UTI is greater in females (80.2%) as compared with males (19.8%) [3]. The prevalence among females during the first year of life was reported to be high in the review report of Almofarreh et al. (2018), which correlates with our results, and may be attributed to the different anatomical structures of the urethra between males and females and other factors such as race, circumcision state, and nutritional state [11]. In a recent study at King Salman Armed Forces Hospital, Tabuk, Saudi Arabia, UTIs were observed more in females (81%) than males (19%), which is in parallel with our study results [12].

Our study’s predominant isolates were *E. coli*, followed by Klebsiella species. These findings confirm reports by other researchers from Saudi Arabia [3]. ESBL-positive *E. coli* was also detected at an increasing rate in our study, which correlates with other reports conducted in Nepal and North of Iran, which showed ESBL rates of 38.9% and 30.5%, respectively [7,13]. A retrospective observational study conducted at a tertiary hospital in Riyadh showed a similar observation that ESBL *E. coli* caused nearly 32% of UTIs in the pediatric age group. [10] In our findings, the following pattern of causative agent was observed *E. coli* > *E. coli ESBL + ve* > *Klebsiella pneumoniae* > *Enterococcus faecalis* > *methicillin-resistant staphylococcus epidermidis* > *coagulase-negative staphylococcus*.

In the present study, antibiotic drug resistance was observed, which is considered a worldwide problem. Our data about the resistance pattern of *E. Coli* for the frequently prescribed medications (sulfamethoxazole/trimethoprim and amoxicillin/clavulanic acid) can be compared with other recent publications. In recent years, several studies specifically investigated the trend of uropathogens resistance in pediatric UTIs in different countries worldwide. The present study could inform treatment by comparing its findings with the most recent data, adding helpful information for clinicians and microbiologists. *E. coli* and *K. pneumoniae* isolates were highly resistant to ampicillin and showed moderate sensitivity towards sulfamethoxazole/trimethoprim and amoxicillin/clavulanic acid antibiotics, similar to other studies reported in Saudi Arabia [3,7]. In agreement with Albalawi et al. (2018), our susceptibility data indicated that all Gram-negative enteric bacilli exhibited a very high prevalence of resistance against ampicillin and sulfamethoxazole/trimethoprim. At the same time, *E. coli* and *K. pneumoniae* were sensitive to gentamicin and norfloxacin [10].

However, in this study, the antibiotic susceptibility testing of the ESBL-positive *E. coli* showed 100% resistance to ampicillin, aztreonam, cefepime, cefoxitin, ceftazidime, ceftriaxone, cefuroxime, and cephalothin. A study conducted by Parajuli et al. in 2017 observed a similar resistance pattern (100%) to ampicillin, aztreonam, cefoxitin, ceftazidime, and ceftriaxone [13]. In our study, carbapenems (100%) were effective in the ESBL-positive, which correlates with the observation conducted by Seo et al. in 2017 [7]. Amikacin (92.31%) showed a similar sensitivity pattern against ESBL-positive *E. coli* in these reports [14]. EAU guidelines state that there are reports of UTIs caused by ESBL-producing Enterobacteriaceae in the pediatric population. In one of the investigations conducted in Turkey, 49% of the children with an age of below 1 year and 38% of those older than one year had ESBL-producing Enterobacteriaceae that were resistant to sulfamethoxazole/trimethoprim in 83%, nitrofurantoin in 18%, quinolones in 47%, and to aminoglycosides in 40% of study population, which comports with the current investigation [15]. A study conducted in Eastern India by Chakraborty et al. in 2015 identified that the Gram-positive Enterococcus species was resistant to erythromycin and gentamicin. A similar pattern was observed in our study [16]. Another study was conducted in a tertiary care hospital in Nepal by Shrestha et al. in 2019. They reported a similar pattern of sensitivity in linezolid (100%) as was observed in our investigation. At the same time, nitrofurantoin (10%) and vancomycin (5%) showed a significantly lower resistance level against *E. faecalis* [17]. A study by Sattari-Maraji et al. in 2019 demonstrated a high frequency of resistance to clindamycin (100%) and erythromycin (98.5%) among *E. faecalis* isolates. In comparison, resistance to ampicillin (7%) was less frequent, whereas in our study, the resistance pattern was 66.67% against clindamycin and erythromycin. Nevertheless, ampicillin showed a moderately high resistance level (42.86%), which is contradictory [18]. A prospective study conducted in Al-Bukayriyah General Hospital, Qassim, Saudi Arabia, detected that *E. faecalis* isolates were susceptible (100%) to teicoplanin and vancomycin but were resistant to erythromycin (49%), tetracycline (38%), and gentamycin (25%), and these findings are consistent with the present study [19].

The first-choice prophylactic antibacterial for UTI are nitrofurantoin and trimethoprim/sulfamethoxazole; in exceptional cases, oral cephalosporin can be used. Recommended antibacterial treatments for cystitis and cystourethritis are oral cephalosporins, nitrofurantoin, trimethoprim/sulfamethoxazole, and amoxicillin/clavulanic acid. Frequently used antibacterial agents for pediatric UTI are parenteral or oral cephalosporins, nitrofurantoin, trimethoprim/sulfamethoxazole, amoxicillin/clavulanic acid, ciprofloxacin, gentamycin, and tobramycin [15]. The current study showed nitrofurantoin is the most prescribed medication in inpatient and outpatient departments, followed by trimethoprim/sulfamethoxazole, amoxicillin/clavulanic acid, cefuroxime, azithromycin, ceftriaxone, and ciprofloxacin, while amikacin, amoxicillin, ampicillin, cefepime, imipenem, and phenoxymethylpenicillin have been demonstrated to be less commonly used and to have a high resistance rate.

## 5. Conclusions

Our study was the first of its kind to research antibiotic resistance in this geographic region, particularly in the pediatric population. There is strong evidence of antibiotic resistance from our previous study conducted on adult populations in the same geographic area and at the same hospital [20]. Henceforth these findings may serve as a guide for clinicians fighting antibiotic resistance. Since children with UTI typically present with ambiguous clinical signs, a urine examination and culture should always be used in the preliminary medical diagnosis of UTI, except when it can be identified with recurring infection and high temperature. Antibiotic resistance can be reduced if antibiotics are used carefully and if their prescription follows the guidelines. Broad-spectrum antibiotics such as cefotaxime, ceftazidime, colistin, levofloxacin, and tigecycline have been proven to be particularly vulnerable against major uropathogens; they should be avoided as empirical therapy and reserved only for complicated UTI. *E. coli* ˃ *K. pneumonia* ˃ *E. faecalis* appears to be a typical causative pediatric uropathogen in this geographical area. According to the findings of our current study, ampicillin, cephalothin, and cefoxitin should be excluded from the management of *E. coli* UTI. Oral cephalosporins, nitrofurantoin, trimethoprim/sulfamethoxazole, and amoxicillin/clavulanic acid are recommended antibacterial treatments for pediatric cystitis and cystourethritis.

## Figures and Tables

**Table 1:** Demographic data of pediatric study population.

	Variables	Number	Percentage (%)	95% CI
Gender	Boys	37	31.36	23.68–40.20
	Girls	81	68.64	59.80–76.32
Age	Newborn (0 to 1 month)	2	1.69	0.46–5.97
	Infant (1 month to 1 year)	23	19.49	13.35–27.55
	Toddler (1 to 3 years)	18	15.25	9.87–22.83
	Preschool (3 to 6 years)	33	27.96	20.66–36.66
	School age 6 to 12 years	29	24.57	17.69–33.06
	Adolescent (12 to 14 years)	13	11.01	6.55–17.94

**Table 2:** Susceptibility pattern of *E. coli* in pediatric UTI patients.

Antibiotics	Test Done (TD)	Resistant *N* (%)	95% CI Resistant	Sensitive *N* (%)	95% CI Sensitive	Test Not Done (ND)
Amikacin	37	2 (5.41)	1.50–17.70	35 (94.59)	82.30–98.50	15
Ampicillin	34	32 (94.12)	80.91–98.37	2 (5.88)	1.63–19.09	18
Amoxicillin/clavulanic acid	14	8 (57.14)	32.59–78.62	6 (42.86)	21.38–67.41	38
Aztreonam	9	1 (11.11)	1.99–43.50	8 (88.89)	56.50–98.01	43
Cefepime	7	2 (28.57)	8.22–64.11	5 (71.43)	35.89–91.78	45
Cefotaxime	1	0 (0.00)	0	1 (100.00)	20.65–100	51
Cefoxitin	13	10 (76.92)	49.74–91.82	3 (23.08)	8.18–50.26	39
Ceftazidime	4	0 (0.00)	0	4 (100.00)	51.01–100.00	48
Ceftriaxone	12	3 (25.00)	8.89–53.23	9 (75.00)	46.77–91.11	40
Cefuroxime	15	7 (46.67)	24.81–69.88	8 (53.33)	30.12–75.19	37
Cephalothin	13	12 (92.31)	66.69–98.63	1 (7.69)	1.37–33.31	39
Ciprofloxacin	22	7 (31.82)	16.36–52.68	15 (68.18)	47.32–83.64	30
Colistin	2	0 (0.00)	0	2 (100.00)	34.24–100	50
Sulfamethoxazole/trimethoprim	45	25 (55.56)	41.18–69.06	20 (44.44)	30.94–58.82	7
Gentamicin	8	1 (12.50)	2.24–47.09	7 (87.50)	52.91–97.76	44
Imipenem	31	2 (6.45)	1.79–20.72	29 (93.55)	79.28–98.21	21
Levofloxacin	2	0 (0.00)	0	2 (100.00)	34.24–100	18
Meropenem	9	1 (11.11)	1.99–43.50	8 (88.89)	56.50–98.01	43
Nalidixic acid	3	1 (33.33)	6.15–79.23	2 (66.67)	20.77–93.85	49
Nitrofurantoin	47	2 (4.26)	1.17–14.25	45 (95.74)	85.75–98.83	5
Norfloxacin	20	5 (25.00)	11.19–46.87	15 (75.00)	53.13–88.81	32
Tazocin	14	2 (14.29)	4.01–39.94	12 (85.71)	60.06–95.99	38
Tigecycline	2	0 (0.00)	0	2 (100.00)	34.24–100	50

TD = respective antibiotic tested against *E. coli*; ND = respective antibiotic was not tested against *E. coli.*

**Table 3:** Susceptibility pattern of ESBL-positive *E. coli* in pediatric UTI patients.

Antibiotics	Test Done (TD)	Resistant *N* (%)	95% CI Resistant	Sensitive *N* (%)	95% CI Sensitive	Test Not Done (ND)
Amikacin	13	1 (7.69)	1.37–33.31	12 (92.31)	66.69–98.63	1
Ampicillin	8	8 (100.00)	67.56–100	0 (0.00)	0–32.44	6
Amoxicillin/clavulanic acid	6	2 (33.33)	9.68–70	4 (66.67)	30–90.32	7
Aztreonam	11	11 (100.00)	74.12–100	0 (0.00)	0–25.88	3
Cefepime	13	13 (100.00)	77.19–100	0 (0.00)	0–22.81	1
Cefoxitin	2	2 (100.00)	34.24–100	0 (0.00)	0–65.76	12
Ceftazidime	3	3 (100.00)	43.85–100	0 (0.00)	0–56.15	11
Ceftriaxone	9	9 (100.00)	70.09–100	0 (0.00)	0–29.91	5
Cefuroxime	3	3 (100.00)	43.85–100	0 (0.00)	0–56.15	11
Cephalothin	2	2 (100.00)	34.24–100	0 (0.00)	0–65.76	12
Ciprofloxacin	11	8 (72.73)	43.44–90.25	3 (27.27)	9.75–56.56	3
Sulfamethoxazole/trimethoprim	9	3 (33.33)	12.06–64.58	6 (66.67)	35.42–87.94	5
Gentamicin	11	7 (63.64)	35.38–84.83	4 (36.36)	15.17–64.62	3
Imipenem	11	0 (0.00)	0–25.88	11 (100.00)	74.12–100	3
Levofloxacin	2	2 (100.00)	34.24–100	0 (0.00)	0–65.76	0
Meropenem	11	0 (0.00)	0–25.88	11 (100.00)	74.12–100	3
Nitrofurantoin	13	0 (0.00)	0–22.81	13 (100.00)	77.19–100	1
Norfloxacin	1	0 (0.00)	0–79.35	1 (100.00)	20.65–100	13
Tazocin	13	1 (7.69)	1.37–33.31	12 (92.31)	66.69–98.63	1

Table legend text. TD = respective antibiotic tested against ESBL-positive *E. coli*; ND = respective antibiotic was not tested against ESBL-positive *E. coli*.

**Table 4:** Susceptibility pattern of *K. pneumoniae* in pediatric UTI patients.

Antibiotics	Test Done (TD)	Resistant *N* (%)	95% CI Resistant	Sensitive *N* (%)	95% CI Sensitive	Test Not Done (ND)
Amikacin	6	0 (0.00)	0–39.03	6 (100.00)	60.97–100	5
Ampicillin	9	9 (100.00)	70.09–100	0 (0.00)	0–29.91	2
Amoxicillin/clavulanic acid	4	2 (50.00)	15–85	2 (50.00)	15–85	7
Aztreonam	3	0 (0.00)	0–56.15	3 (100.00)	43.85–100	8
Cedoxitine	2	0 (0.00)	0–65.76	2 (100.00)	34.24–100	9
Cefoxitin	1	1 (100.00)	20.65–100	0 (0.00)	0–79.35	10
Ceftriaxone	6	2 (33.33)	9.68–70	4 (66.67)	30–90.32	5
Cefuroxime	2	2 (100.00)	34.24–100	0 (0.00)	0–65.76	8
Cephalothin	3	3 (100.00)	43.85–100	0 (0.00)	0–56.15	8
Ciprofloxacin	3	0 (0.00)	0–56.15	3 (100.00)	43.85–100	7
Sulfamethoxazole/trimethoprim	11	7 (63.64)	35.38–84.83	4 (36.36)	15.17–64.62	0
Gentamicin	1	0 (0.00)	0–79.35	1 (100.00)	20.65–100	10
Imipenem	6	0 (0.00)	0–39.03	6 (100.00)	60.97–100	4
Levofloxacin	2	0 (0.00)	0–65.76	2 (100.00)	34.24–100	3
Meropenem	5	1 (20.00)	3.62–62.45	4 (80.00)	37.55–96.38	7
Nitrofurantoin	10	2 (20.00)	5.67–50.98	8 (80.00)	49.02–94.33	1
Norfloxacin	5	1 (20.00)	3.62–62.45	4 (80.00)	37.55–96.38	6
Tazocin	5	2 (40.00)	11.76–76.93	3 (60.00)	23.07–88.24	6
Tobramycin	1	1 (100.00)	20.65–100	0 (0.00)	0–79.35	8

TD = respective antibiotic tested against *K. pneumoniae*; ND = respective antibiotic was not tested against *K. pneumoniae.*

**Table 5:** Susceptibility pattern of *E. faecalis* in pediatric UTI patients.

Antibiotics	Test Done (TD)	Resistant *N* (%)	95% CI Resistant	Sensitive *N* (%)	95% CI Sensitive	Test Not Done (ND)
Ampicillin	7	3 (42.86)	15.82–74.95	4 (57.14)	25.05–84.18	0
Cefepime	1	1 (100.00)	20.65–100	0 (0.00)	0–79.35	6
Cefoxitin	2	1 (50.00)	9.45–90.55	1 (50.00)	9.45–90.55	4
Cephalothin	1	0 (0.00)	0–79.35	1 (100.00)	20.65–100	6
Ciprofloxacin	3	0 (0.00)	0–56.15	3 (100.00)	43.85–100	4
Clindamycin	3	2 (66.67)	20.77–93.85	1 (33.33)	6.15–79.23	4
Sulfamethoxazole/trimethoprim	4	1 (25.00)	4.56–69.94	3 (75.00)	30.06–95.44	3
Erythromycin	6	4 (66.67)	30–90.32	2 (33.33)	9.68–70	1
Gentamicin	4	2 (50.00)	15–85	2 (50.00)	15–85	3
Imipenem	3	1 (33.33)	6.15–79.23	2 (66.67)	20.77–93.85	4
Linazolid	2	0 (0.00)	0–65.76	2 (100.00)	34.24–100	5
Moxifloxacin	3	0 (0.00)	0–56.15	3 (100.00)	43.85–100	4
Nitrofurantoin	3	0 (0.00)	0–56.15	3 (100.00)	43.85–100	4
Teicoplanin	3	0 (0.00)	0–56.15	3 (100.00)	43.85–100	4
Tetracycline	2	2 (100.00)	34.24–100	0 (0.00)	0–65.76	5
Vancomycin	3	0 (0.00)	0–56.15	3 (100.00)	43.85–100	4

Table legend text. TD = respective antibiotic tested against *Enterococci faecalis*; ND = respective antibiotic was not tested against *Enterococci faecalis*.

## Data Availability

The data presented in this study are available on request from the corresponding author. The data are not publicly available due to hospital’s internal policy.
